# Glycated hemoglobin in immediate postpartum, overweight, and pre-gestational arterial hypertension: effect on fasting glycemia one year postpartum

**DOI:** 10.61622/rbgo/2026rbgo150

**Published:** 2026-09-23

**Authors:** Shirley Vidal Amorim, Luiz Gonzaga Ribeiro Silva-Neto, Giovana Longo-Silva, Jonas Augusto Cardoso da Silveira, Edward Araujo Júnior, Lucas Almeida das Chagas, Risia Cristina Egito de Menezes

**Affiliations:** 1 Universidade Federal de Alagoas Maceio AL Brazil Universidade Federal de Alagoas, Maceio, AL, Brazil.; 2 Universidade Federal de São Paulo Escola Paulista de Medicina São Paulo SP Brazil Escola Paulista de Medicina, Universidade Federal de São Paulo, São Paulo, SP, Brazil.; 3 Universidade Federal do Paraná Curitiba PR Brazil Universidade Federal do Paraná, Curitiba, PR, Brazil.; 4 Universidade Municipal de São Caetano do Sul São Caetano do Sul SP Brazil Universidade Municipal de São Caetano do Sul, São Caetano do Sul, SP, Brazil.

**Keywords:** Diabetes, Pregnancy, Hypertension, Overweight, Glycated hemoglobin, Postpartum period, Diabetes mellitus, type 2

## Abstract

**Objective:**

To assess the relationship between overweight, pre-gestational hypertension, and glycated hemoglobin (HbA1c) levels in the immediate postpartum and fasting glycemia (FG) in low-income women one year after birth.

**Methods:**

This prospective cohort study assessed women who gave birth between February and August 2017 with gestational age > 35 weeks. Interviews and anthropometric evaluations were conducted within the first 24 hours after delivery. Pre-gestational anthropometric nutritional status was assessed based on maternal height measurement. Blood samples were collected after delivery and over the next year, and HbA1c ≥ 5.7% and FG > 100 mg/dL indicated a high risk of type 2 diabetes mellitus (T2DM). A generalized linear mixed-effects regression model was applied.

**Results:**

Among the 130 women included, 28.0% and 2.3% were overweight and had pre-pregnancy hypertension, respectively; 20.0% had altered HbA1c in the immediate postpartum. As for FG, 4.6% and 14.8% were at risk of T2DM at the 6th and 12th months postpartum, respectively. The mean ± standard deviation of FG among the evaluated women was 77.9 ± 10.98 mg/dL after 6 months and 81.8 ± 15.49 mg/dL after one year of follow-up. In the model adjusted for age and schooling, the effect of overweight and pre-pregnancy hypertension was observed, as well as altered HbA1c in the immediate postpartum period, on the elevation of FG, increasing the risk of T2DM.

**Conclusion:**

Altered HbA1c in the immediate postpartum period, pre-gestational overweight, and pre-gestational hypertension are risk factors for T2DM among low-income women, indicating the need to monitor postpartum glycemia.

## Introduction

Diabetes mellitus is a metabolic disorder characterized by high plasma glucose levels,^(1)^ with type 2 (T2DM) being the most prevalent worldwide.^(2)^ Gestational diabetes mellitus (GDM) is characterized by glucose intolerance diagnosed during pregnancy. Although it is not considered a direct cause of diabetes mellitus, it represents a significant risk factor for the future development of type 2 diabetes mellitus (T2DM).^(3)^ Women diagnosed with gestational diabetes are at increased risk of developing persistent health complications in the postpartum period, including impaired glycemic control. Furthermore, as described in the literature, there is an increased likelihood of progression to T2DM in the short- and long-term.^(3)^

In addition to GDM, it is worth noting that the presence of unfavorable pre-pregnancy conditions, such as overweight and hypertension, is also associated with an increased cardiometabolic risk and may contribute to poorer glycemic control in the postpartum period.^(4)^ In this context, the postpartum period represents a critical window for the early identification of metabolic changes and the implementation of preventive strategies to reduce the risk of developing T2DM.^(5)^

However, follow-up monitoring of glycemic changes remains inadequate, particularly in low- and middle-income countries, where limited access to health services, insufficient counseling, and challenges of adjusting to motherhood contribute to low adherence to follow-up care.^(6)^ These challenges highlight the need for simple, accessible, and early screening strategies, which should ideally be implemented in the immediate postpartum period, when access to healthcare services is still assured. In this context, glycated hemoglobin (HbA1c), which reflects average blood glucose levels over the past two to three months,^(7,8)^ has been widely used in the diagnosis and monitoring of T2DM and may constitute a practical alternative for screening in the postpartum period, especially when fasting blood tests are not feasible.

In the immediate postpartum period, the use of HbA1c may be particularly advantageous in settings with low adherence to follow-up care, as it does not require fasting and can be performed before hospital discharge. Despite its potential, the use of HbA1c as an indicator of glycemic changes during this period remains underexplored, especially in socially vulnerable populations.

In Brazil, where socioeconomic inequalities may further limit access to postpartum care, it is particularly important to investigate viable strategies for the early identification of women at risk for T2DM, with a view to strengthening primary health care initiatives. In this regard, it is necessary to deepen our understanding of the utility of HbA1c, measured in the immediate postpartum period and in association with pre-pregnancy risk factors, as a tool for identifying women with a higher probability of developing subsequent glycemic abnormalities.

This study aimed to evaluate whether pre-gestational overweight, pre-gestational hypertension, and postpartum HbA1c levels are associated with fasting blood glucose up to one year postpartum in low-income women from Northeast Brazil.

## Methods

It was a prospective cohort study based on data from the birth cohort "Health, Nutrition, and Child Development" (SAND), designed to investigate aspects of maternal-child health, nutrition, development, and feeding practices from birth to one year postpartum.

The study took place in the municipality of Rio Largo, located in the state of Alagoas, Northeast Brazil, a federative unit with one of the lowest human development indices (HDI) in the country. This municipality was chosen because its historical HDI is similar to the state's HDI.^(9)^

All women who gave birth at the maternity ward of the only hospital in Rio Largo, Alagoas, between February and August 2017, with gestational age > 35 weeks, were eligible. This maternity ward is known for attending to low-risk pregnant women and having an exclusively public administrative regime. The sample was non-probabilistic and convenience-based, considering the consecutiveness of all eligible births.

The eligibility criteria included mothers with permanent residency in the municipality, gestational age above 35 weeks, biochemical tests requested during follow-up, and absence of language or speech disorders, human immunodeficiency virus (HIV/AIDS), and clinical conditions that would prevent breastfeeding, the initiation of breastfeeding, or the introduction of food. Out of the 284 eligible births, 240 women agreed to participate in the SAND cohort, of which 130 underwent at least one biochemical evaluation.

Data collection took place in 2017 and 2018, with 12 months of follow-up. A total of 240 women gave birth at the maternity hospital during the study period. Among these, 170 underwent HbA1c assessment in the immediate postpartum period at the maternity hospital. Fasting glucose measurements were obtained from 130 women at 6 months postpartum and from 142 women at 12 months postpartum. For the analytical model, only participants with complete data across the three time points were included, resulting in a final analytical sample of 130 women. The remaining 110 participants were excluded due to loss to follow-up, resulting in incomplete biochemical data and precluding their inclusion in the analytical strategy. No data imputation was performed. Losses to follow up across the study period are presented in figure 1.

**Figure 1:**
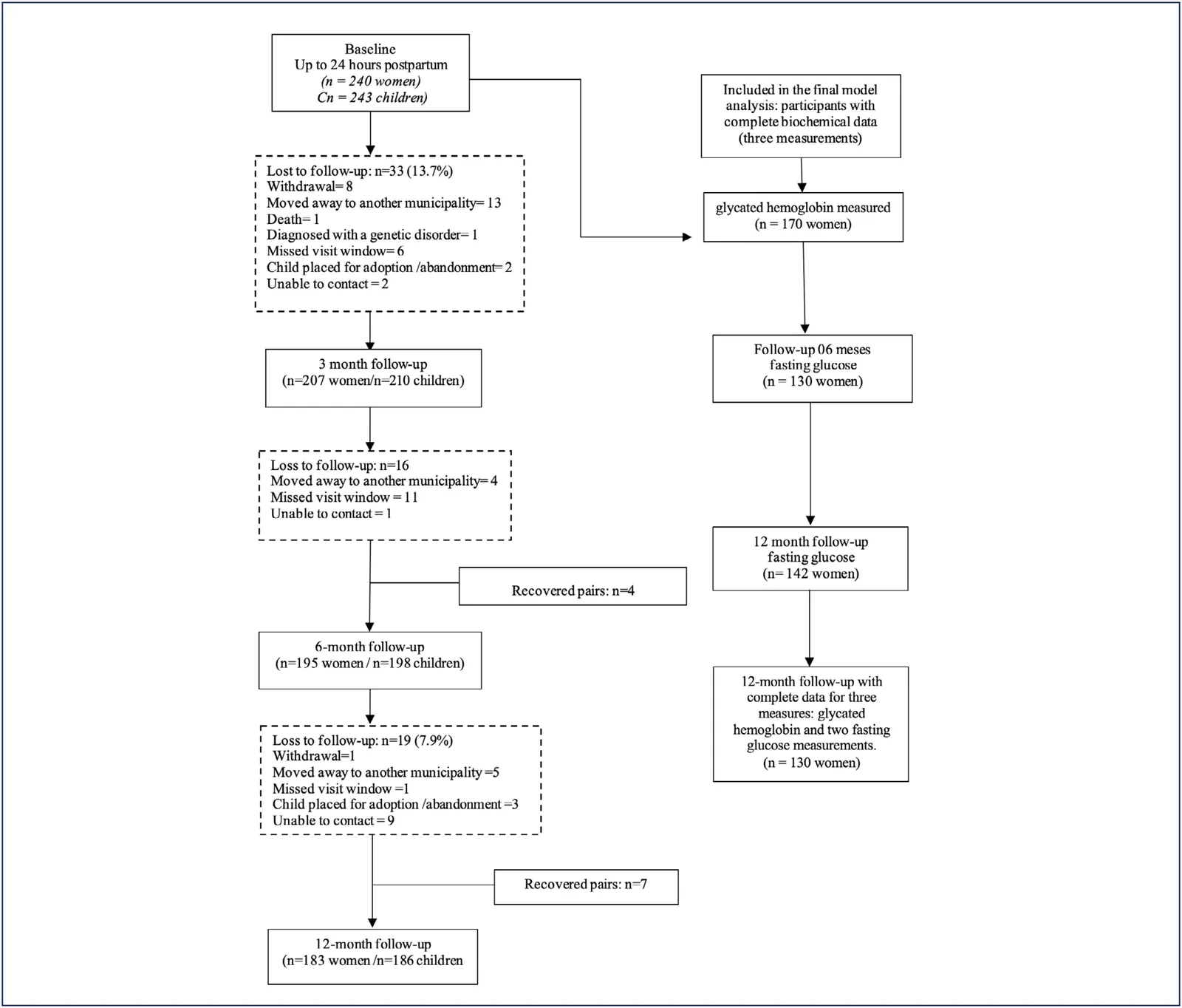
Study population comprising women from the cohort

Missing data varied across variables, with complete data for age and pre-gestational arterial hypertension (n=130), and partial missingness for years of study (n=0), poverty status (n=26), pre-pregnancy overweight (n=30), and risk of T2DM (n=15). Missing data for sample characterization variables are detailed in table 1. The interviews and assessments were conducted by trained nutritionists, supported by a field supervisor, using structured, standardized, and pre-coded forms on demographic and socioeconomic information. Data entry and validation took place within 24 hours after collecting independent pairs, using the Epi-Info 3.5.4 software (Centers for Disease Control and Prevention [CDC], Atlanta, GA, USA).

**Table 1:** Characteristics of women according to the study's inclusion and exclusion criteria

		Included (n=130)	Excluded (n= 110)	p-value
		n(%)	n(%)	
Age (n=130)				
	< 20 years	42(32.3)	30(27.3)	0.396
	≥ 20 years	88(67.7)	80(72.7)	
Years of study (n=130)				
	≤ 8 years	47(36.2)	54(49.1)	0.043
	> 8 years	83(63.8)	56(50.9)	
Poverty status (n=104)				
	Yes	87(83.6)	75(91.5)	0.115
	No	17(16.4)	7(8.5)	
Overweight pre-pregnancy (n=100)				
	Yes	28(28.0)	28(34.2)	0.371
	No	72(72.0)	54(65.8)	
Pre-gestational arterial Hypertension (n=130)				
	Yes	3(2.3)	2(1.8)	0.791
	No	127(97.7)	108(98.2)	
Risk of type 2 diabetes mellitus (n=115)^**^				
	Yes	23(20.0)	10(18.2)	0.779
	No	92(80.0)	45(81.8)	

Women were excluded from this study due to the absence of biochemical parameters

* p-value from the Chi-Square test; a Classification of risk of type 2 diabetes mellitus based on glycated hemoglobin values ≥ 5.7%

The following variables were analyzed: age in years (< 20 years; ≥ 20 years), education in years of study (≤ 8 years; > 8 years of study), and monthly per capita family income (considering the cutoff points: for poverty -R$ < 499.00; and above poverty - ≥ R$ 499.00).^(10)^ The previous medical diagnosis of arterial hypertension (no; yes) was assessed through self-report.

Pre-gestational anthropometric data, including weight and height, were obtained from the "Pregnant Woman's Health Card". When these records were unavailable, the information was self-reported by the pregnant woman, in the following order of priority. Pre-gestational overweight was defined as a body mass index (BMI) ≥ 25 kg/m^2^, in accordance with the World Health Organization (WHO) recommendations and adopted by the Brazilian Ministry of Health.^(11)^

Blood was collected within seven days after delivery (n=170) for biochemical evaluation of HbA1c. Values were analyzed using the cutoff points established by the American Diabetes Association (ADA), which considers HbA1c levels ≥ 5.7% indicative of risk for T2DM postpartum.^(4,5)^

Subsequently, new blood samples were collected to assess fasting glucose levels at the 6th (n=130) and 12th (n=142) months postpartum. Fasting glucose levels ≥100 mg/dL, which indicate impaired fasting glucose rather than diabetes, were used as an indicator of increased risk for T2DM. An accredited laboratory conducted all blood collections and subsequent biochemical assessments in accordance with recommended protocols for good laboratory practice in sample collection and analysis.

A descriptive analysis was conducted to characterize the study population and to describe the prevalence of T2DM risk according to the analyzed characteristics. The characteristics of included (n=130) and excluded (n=110) women were compared using Pearson's chi-square test and Fisher's exact test. To describe fasting glucose levels according to postpartum time (6th and 12th months), the mean and standard deviation (SD) of fasting glucose, along with the proportion of women at risk of T2DM, were presented.

To assess the adequacy of the statistical model, we analyzed the normality of the residuals using the Shapiro-Wilk test, multicollinearity using the variance inflation factor (VIF), and heteroscedasticity using the Breusch-Pagan-Godfrey test. The values found indicated that there was no multicollinearity or heteroscedasticity and that the model was well-adjusted. We used Cook's distance to detect leverage points and did not identify any observations with disproportionate influence on the estimated parameters.

A mixed-effects multiple linear regression model was developed to assess fasting blood glucose levels (outcome variable). The model considers fixed effects (time, postpartum HbA1c, nutritional status, and pre-gestational arterial hypertension) and random effects (individual and time) (exposure variables) to estimate the variation of coefficients among individuals.

The statistical modeling adopted has two components: the intra-individual (longitudinal change described by the model with a cluster-population intercept and slope) and between-individual (variation in individual intercept and slope). The strategy used allows for analyses based on data from individuals with temporally unbalanced measurements (obtained at different time intervals), incorporating the dependence and correlation structure of errors. For this modeling, we used the unstructured covariance matrix. Regression coefficients were estimated using the Maximum Likelihood method.

A mixed-effects linear regression model was used to assess fasting blood glucose levels (outcome variable). The model included fixed effects for postpartum time (6th vs 12th month), pre-gestational overweight, pre-gestational arterial hypertension, and risk of T2DM based on immediate postpartum HbA1c. Random effects were specified for each individual, allowing both the intercept and the slope for postpartum time to vary. An unstructured covariance matrix was used for the random effects, and robust standard errors clustered by participants accounted for within-subject correlation. All variables included in the analyses were complete, with no missing values, and regression coefficients were estimated using the Maximum Likelihood method. The model can be expressed as: Glucoseij=β0+β1(Timeij)+β2(Overweighti+ β3(Hypertensioni)+β4(HbA1ci)+u0i+u1i(Timeij)+εij, where u0i represents the subject-specific random intercept, u1i represents the subject-specific random slope for postpartum time, and εij represents the residual error term for observation j within individual i. This specification allows the model to handle temporally unbalanced measurements and capture both within- and between-individual variability.

Beta coefficients and their 95% confidence intervals (95% CI) were estimated using simple (unadjusted) and multiple (adjusted) mixed-effects linear regression analyses to examine the associations between postpartum time, pre-gestational BMI, pre-gestational hypertension, and HbA1c immediately postpartum with fasting glucose levels in socioeconomically vulnerable women, adjusting for age and education. Beta values represent the mean change in fasting glucose associated with a one-unit change in each independent variable. Confidence intervals for β that do not include zero indicate a statistically significant association (p ≤ 0.05). Analyses were conducted using Stata/SE 13.0 software (StataCorp LP, College Station, TX, USA), with a significance level of 0.05.

The larger study, the SAND cohort, from which this work derives, was approved by the Research Ethics Committee of the Federal University of Alagoas (CAAE: 55483816.9.0000.5013, approval number 1.541.581). The study was conducted following the guidelines of the Helsinki Declaration. All participants provided written informed consent to participate in the study.

## Results

A total of 130 women were included, with losses and exclusions presented in figure 1. The characteristics of the women included and excluded in the study are presented in table 1. No evidence of selection bias was observed for most of the analyzed variables.

Among the included women, 67.7% were aged ≥ 20 years, 63.8% had more than 8 years of education, and 83.6% were classified as living in poverty. Regarding the pre-gestational period, we observed a prevalence of 28% for overweight and 2.3% for a previous medical diagnosis of arterial hypertension. In the immediate postpartum period, 20% were at risk for T2DM. Regarding fasting blood glucose, 4.6% and 14.8% of the women presented a risk of T2DM at 6 and 12 months postpartum, respectively. The mean fasting blood glucose among the women evaluated was 77.9 ± 10.98 mg/dL (SD) at 6 months postpartum and 81.8 ± 15.49 mg/dL (SD) after one year of follow-up (Table 2).

**Table 2:** Prevalence of diabetes mellitus risk, mean, and standard deviation of fasting glycemia, among women one year postpartum

	Risk of type 2 diabetes mellitus*	Fasting glucose concentration (mg/dL)	
	n(%)	Mean	± SD
6th month postpartum*	6(4.6)	77.9	10.98
12th month postpartum*	21(14.8)	81.8	15.49

* Risk of type 2 diabetes mellitus is considered when taking into account fasting glucose values (fasting glucose ≥100 mg/dL)

The results of the multiple linear regression model with fasting glucose levels as the dependent variable are shown in table 3. Considering the raw values, factors associated with higher glucose levels among low-income women one year after childbirth were: time from delivery (β: 3.89; 95% CI [1.69; 7.10]), pre-gestational overweight (β: 7.75; 95% CI [3.90; 11.60]), previous diagnosis of arterial hypertension (β: 14.60; 95% CI [4.53; 24.66]), and changes in postpartum HbA1c levels (β: 5.88; 95% CI [1.66; 10.10]).

**Table 3:** Factors associated with changes in fasting blood glucose among women one year postpartum, according to a mixed-effects generalized linear regression model

		β	Crude	p-value	Adjusted value		
			95% CI		β	95% CI	p-value
Stage (postpartum time)							
	6th month	Ref			Ref		
	12th month	3.89	[1.69; 7.10]	0.017	3.16	[-0.38; 6.70]	0.080
Nutritional status (pre-pregnancy)							
	Eutrophic	Ref			Ref		
	Overweight	7.75	[3.90; 11.60]	< 0.001	7.38	[2.69; 12.07]	0.002
Diagnosis of arterial hypertension (pre-gestational)							
	No	Ref			Ref		
	Yes	14.60	[4.53; 24.66]	0.004	16.23	[5.53; 26.84]	0.003
Type 2 diabetes mellitus (immediate postpartum)*							
	No risk	Ref			Ref		
	At risk	5.88	[1.66; 10.10]	0.006	3.98	[1.20; 7.76]	0.039

* Classification of risk of type 2 diabetes mellitus based on glycated hemoglobin values ≥ 5.7%. 95%CI: 95% Confidence Interval; Model controlled for age and schooling

After adjusting the model for age and education, women who were overweight before pregnancy had, on average, a fasting blood glucose level 7.38 mg/dL higher than women with normal weight (95% CI [2.69; 12.07]), representing a modest but clinically significant increase. Those with a prior diagnosis of hypertension had a fasting blood glucose level 16.23 mg/dL higher (95% CI [5.53; 26.84]), a substantial and clinically significant difference; however, the prevalence of pre-gestational hypertension was low (2.3%), which may contribute to the instability in the magnitude of the estimated effect. Furthermore, women classified as at risk for T2DM based on HbA1c immediately after delivery had a 3.98 mg/dL higher fasting blood glucose level (95% CI [1.20; 7.76]), highlighting the impact of glycemic changes in the early postpartum period on subsequent fasting blood glucose levels (Table 3).

## Discussion

Our findings indicate that higher fasting glucose levels one year after delivery were associated with (i) pre-gestational overweight, (ii) pre-gestational arterial hypertension, and (iii) elevated HbA1c levels in the immediate postpartum period. These findings underscore the importance of early metabolic risk factors in the progression to T2DM in the postpartum period.

As the trend of overweight in the Brazilian population rises,^(12)^ women of reproductive age represent a particularly vulnerable group. Overweight before pregnancy is consistently associated with adverse metabolic outcomes, including persistence of overweight and increased risk of T2DM after childbirth.^(13-15)^ In this context, pre-pregnancy overweight may contribute to unfavorable glycemic trajectories in the postpartum period, reinforcing its role as an early marker of metabolic risk. Furthermore, it is associated with adverse nutritional outcomes for the fetus, from the intrauterine stage through the postnatal period, and may extend into early childhood.^(16,17)^

Similar to overweight, arterial hypertension should not be interpreted as a direct causal factor of T2DM, but rather as a comorbidity that shares common pathophysiological mechanisms and contributes to increased cardiometabolic risk.^(18)^ It can be observed that women with hypertension prior to pregnancy may be more likely to experience adverse outcomes during the postpartum period.^(19,20)^ Our findings suggest that women with pre-gestational arterial hypertension presented higher fasting glucose levels after childbirth; however, this result should be interpreted with caution, given the low prevalence of hypertension in the sample, which may affect the stability of the estimates.

HbA1c is a relevant indicator in this context, as it reflects average blood glucose levels and is widely used in the diagnosis and monitoring of T2DM.^(21,22)^ In the hospital setting, particularly before discharge during the immediate postpartum period, measuring HbA1c can provide a valuable opportunity to identify women at increased risk early.^(23)^ In the present study, altered HbA1c levels in the immediate postpartum period were associated with higher fasting blood glucose levels up to one year after delivery, suggesting its potential as an early marker of future glycemic changes.

However, the interpretation of HbA1c in the immediate postpartum period should be approached with caution. Physiological changes associated with pregnancy and childbirth, including anemia, blood loss, and alterations in erythrocyte turnover, can influence its levels and compromise its accuracy during this period.^(7)^ In this study, certain conditions, such as overweight and hypertension, which are components of cardiometabolic syndrome, were evaluated.^(24)^ However, metabolic syndrome, as a clinical condition, was not directly investigated; therefore, its interpretation in this context should be approached with caution.

We found a prevalence of approximately 15% of T2DM risk (fasting glucose) 12 months after childbirth among the women evaluated. This finding highlights the persistence of metabolic risk after pregnancy, particularly among socially vulnerable women. The Brazilian Ministry of Health advocates, among the healthcare protocols, for the diagnosis of diabetes mellitus in the primary healthcare network within the scope of Brazil's Unified Health System.^(25)^ However, the measurement of HbA1c in the postpartum period and throughout the first year after childbirth has not been established, so this test is not included in the screening items used in primary healthcare for the control of T2DM.^(26)^

Our findings suggest that measuring HbA1c in the immediate postpartum period may help identify women at increased risk of subsequent glycemic alterations, particularly in settings with low adherence to follow-up. However, these results should be interpreted with caution, given the sample size and the single-municipality study design. They should be regarded as exploratory rather than sufficient to support modifications to screening protocols or health policies.

It's important to highlight that the costs associated with managing T2DM, arterial hypertension, and overweight, including hospitalization, outpatient procedures, and medication expenses covered by Brazil's Unified Health System, are known to be higher than the costs of prevention.^(27)^ Therefore, early identification of women at increased risk may contribute to the implementation of preventive strategies and the reduction of long-term complications.

This study has several strengths, including its longitudinal design with data collected over a year and the use of multiple biochemical parameters. The use of repeated biochemical measurements over time strengthens the assessment of glycemic changes in the postpartum period. Among the study limitations, we note the relatively small size of the evaluated population, which did not allow for precise estimates for the proposed regression model, as evidenced by the wide confidence intervals obtained. Additionally, some loss to follow-up occurred, and the study population was limited to low-income women from a single municipality, which may restrict the external validity of our findings. Biochemical parameters during pregnancy were unavailable; however, this limitation was mitigated by evaluating HbA1c in the immediate postpartum period, which inferred that the glucose measurement reflected the last trimester of pregnancy. Furthermore, as pre-gestational weight data were obtained from the pregnant woman's health record or self-reporting, measurement bias cannot be ruled out.

## Conclusion

In conclusion, we observed that women were at risk of or presented altered fasting glucose levels suggestive of increased risk for T2DM after childbirth. Higher fasting glucose levels during the first postpartum year were associated with pre-gestational overweight, pre-gestational arterial hypertension, and altered HbA1c levels in the immediate postpartum period. These findings suggest that HbA1c testing shortly after delivery may help identify women at increased risk of subsequent glycemic alterations, particularly in low-income settings. However, given the relatively small sample size and the single-municipality design, these results should be considered exploratory. Further studies involving larger, more diverse populations are needed to confirm these associations and better understand the potential role of postpartum HbA1c in strategies for the prevention and early detection of T2DM.

## Data Availability

The research data are described in the article presented.

## References

[1] Khan RM, Chua ZJ, Tan JC, Yang Y, Liao Z, Zhao Y (2019). From pre-diabetes to diabetes: diagnosis, treatments and translational research. Medicina (Kaunas).

[2] Ornoy A, Becker M, Weinstein-Fudim L, Ergaz Z (2021). Diabetes during pregnancy: a maternal disease complicating the course of pregnancy with long-term deleterious effects on the offspring. a clinical review. Int J Mol Sci.

[3] Vounzoulaki E, Khunti K, Abner SC, Tan BK, Davies MJ, Gillies CL (2020). Progression to type 2 diabetes in women with a known history of gestational diabetes: systematic review and meta-analysis. BMJ.

[4] Sociedade Brasileira De Diabetes (2023). Diretriz da Sociedade Brasileira de Diabetes - EDIÇÃO.

[5] American Diabetes Association (2019). 2. Classification and Diagnosis of Diabetes: Standards of Medical Care in Diabetes-2019. Diabetes Care.

[6] Balaji B, Ranjit Mohan A, Rajendra P, Mohan D, Ram U, Viswanathan M (2019). Gestational diabetes mellitus postpartum follow-up testing: challenges and Solutions. Can J Diabetes.

[7] Guo W, Zhou Q, Jia Y, Xu J (2019). Increased levels of glycated hemoglobin a1c and iron deficiency anemia: a review. Med Sci Monit.

[8] Lu W, Hu C (2022). Molecular biomarkers for gestational diabetes mellitus and postpartum diabetes. Chin Med J (Engl).

[9] Instituto Brasileiro de Geografia e Estatística (IBGE) (2019). Estimativas da população residente no Brasil e unidades da Federação com data de referência em 1o de julho de 2019.

[10] Banco Mundial (2022). Relatório de Pobreza e Equidade no Brasil. Mirando o futuro após duas crises. Sumário Executivo.

[11] World Health Organization (WHO) (1995). Expert Committee on Physical Status: The Use and Interpretation of Anthropometry Physical status.

[12] Silva LE, Oliveira MM, Stopa SR, Gouvea EC, Ferreira KR, Santos RO (2021). Temporal trend of overweight and obesity prevalence among Brazilian adults, according to sociodemographic characteristics, 2006-2019. Epidemiol Serv Saude.

[13] Flores TR, Nunes BP, Miranda VI, Silveira MF, Domingues MR, Bertoldi AD (2020). [Gestational weight gain and postpartum weight retention: data from the 2015 birth cohort in Pelotas, Rio Grande do Sul State, Brazil]. Cad Saude Publica.

[14] Santos MM, Baião MR, de Barros DC, Pinto AA, Pedrosa PL, Saunders C (2012). [Pre-pregnancy nutritional status, maternal weight gain, prenatal care, and adverse perinatal outcomes among adolescent mothers]. Rev Bras Epidemiol.

[15] González-Plaza E, Bellart J, Martínez-Verdú MÁ, Arranz Á, Luján-Barroso L, Seguranyes G (2022). Pre-pregnancy overweight and obesity prevalence and relation to maternal and perinatal outcomes. Enferm Clin (Engl Ed).

[16] Oliveira MM, Santos EE, Bernardino ÍM, Pedraza DF (2022). [Factors associated with nutritional status of children under five years old from Paraíba, Brazil]. Cienc Saude Colet.

[17] Oliveira AC, Pereira LA, Ferreira RC, Clemente AP (2018). [Maternal nutritional status and its association with birth weight in high-risk pregnancies]. Cienc Saude Colet.

[18] Teck J (2022). Diabetes-Associated Comorbidities. Prim Care.

[19] Butts SJ, Huber LR (2022). Pre-pregnancy diabetes, pre-pregnancy hypertension and prenatal care timing among women in the United States, 2018. Matern Child Health J.

[20] Lauder L, Mahfoud F, Azizi M, Bhatt DL, Ewen S, Kario K (2023). Hypertension management in patients with cardiovascular comorbidities. Eur Heart J.

[21] Eyth E, Zubair M, Naik R (2026). Hemoglobin A1C. 2025.

[22] Incani M, Sentinelli F, Perra L, Pani MG, Porcu M, Lenzi A (2015). Glycated hemoglobin for the diagnosis of diabetes and prediabetes: diagnostic impact on obese and lean subjects, and phenotypic characterization. J Diabetes Investig.

[23] Malta DC, Duncan BB, Schmidt MI, Machado IE, da Silva AG, Bernal RT (2019). Prevalence of diabetes mellitus as determined by glycated hemoglobin in the Brazilian adult population, National Health Survey. Rev Bras Epidemiol.

[24] Lima MD, Melo AS, Sena AS, Barros VO, Amorim MM (2019). Metabolic syndrome in pregnancy and postpartum: prevalence and associated factors. Rev Assoc Med Bras.

[25] Brasil. Ministério da Saúde. Secretaria de Atenção à Saúde. Departamento de Atenção Básica (2013). (2013) Estratégias para o cuidado da pessoa com doença crônica: diabetes mellitus / Ministério da Saúde, Secretaria de Atenção à Saúde, Departamento de Atenção Básica.

[26] Organização Pan-Americana da Saúde (OPAS). Ministério da Saúde. Federação Brasileira das Associações de Ginecologia e Obstetrícia. Sociedade Brasileira de Diabetes (2016). Rastreamento e diagnóstico de diabetes mellitus gestacional no Brasil.

[27] Nilson EA, Andrade RD, de Brito DA, de Oliveira ML (2020). [Costs attributable to obesity, hypertension, and diabetes in the Unified Health System, Brazil, 2018]. Rev Panam Salud Publica.

